# A Hospital's Water Supply

**Published:** 1922-07

**Authors:** W. H. Diamond, Leonard D. Rea

**Affiliations:** *Chairman*. King Edward VII. Hospital, Cardiff; *Secretary*. King Edward VII. Hospital, Cardiff


### A Hospital's Water Supply.

*To the Editor of* The Hospital and Health Review.

Sir,—The reference recently made to the increased consumption of water at the King Edward VII. Hospital, Cardiff, was carefully noted at the time by the hospital authorities, who were prepared to give reasons for the increased consumption, but delayed communicating with the Press until the Corporation had considered the proposal to increase their contribution to the hospital. Now that that has been dealt with, it is only fair to the hospital that the facts should be made known—especially so in view of the fact that your publication has referred to the local report. A comparative statement presented by the Waterworks Engineer showed a considerable increase in consumption during 1921 in comparison with 1919. The great scheme of extensions, initiated by the late Colonel Bruce Vaughan, reached completion towards the end of 1919, bringing into commission new wards and departments, also a new and greatly extended kitchen, new boilers, and a refrigerating plant. These additions, in themselves, will account for a very large increase in consumption between the periods quoted. In addition, the increase in the work of the hospital generally since 1919 has been very heavy ; more than 500 additional in-patients have been treated during 1921, as compared with 1919, and the number of major operations performed in that year shows an increase of nearly 30 per cent. The work at the Maternity Hospital was three times greater in 1921 than in 1919, and in considering this matter in terms of beds or patients, one has to remember the additional staff necessary, also the pressure on all the various activities of the hospital, including the massage and therapeutic baths department. Comparing the two years quoted, the work at the laundry (which is very considerable) has increased by at least 30 per cent.

It is also of interest to remember that there was a four months' drought during 1919, and that the water supply was actually restricted from August 16 to August 28, following upon the grave official warnings from the Waterworks Department during the whole of the summer. Further, during the year 1919 the work at the hospital was at its lowest ebb, impeded as it was by extensive building operations and by slackness consequent upon the termination of the war—our hospital staff being only gradually restored to civil work. On the other hand, in 1921 work has been carried out in the fullest possible manner, with every curative activity at high pressure ; the Welsh National Medical School as well as the new and extended casualty department having been opened before the termination of the year, all these activities increasing the personnel of the institution, and thus increasing the consumption of water during the period under review.

For and on behalf of the Board of Management,

W. H. Diamond,
*Chairman.*

Leonard D. Rea,
*Secretary.*

King Edward VII. Hospital, Cardiff.

